# Death from Inhalation of Ether

**Published:** 1873-11-15

**Authors:** 


Death from the Inhalation of Ether.—The *British Medical Journal* records a case of death apparently directly resulting from the inhalation of *ether*. A boy, fourteen years of age, of strumous diathesis, who had suffered from repeated attacks of corneitis, was admitted to the Royal South Hants Infirmary under care of Dr. Lake. It was thought best to perform iridectomy, and for this purpose ether was administered. Half an ounce of ether was first poured on a sponge contained in a cone, and closely applied to the mouth and nose. After a few minutes' inhalation three drachms more were poured on the sponge. Shortly after commencing to inhale this second quantity he began to struggle violently, getting at length into a state bordering on opisthotonos, his face being intensely scarlet. It was then noted that his pulse had become very feeble. The ether was at once discontinued, when the pulse having improved Dr. Lake operated, no more ether being administered. At the close of the operation, which occupied only a few seconds in its performance, and before the eye could be bandaged, the pulse became imperceptible, the breathing was suspended, and the countenance livid. All the usual means were resorted to to endeavor to resuscitate the patient, but without success. At the necropsy the brain was found to be healthy, and not congested. The right cavities of the heart were full of dark fluid blood, but the left cavities contained only about a drachm of similar fluid. The valves were healthy. The muscular structures, although somewhat flabby, presented no decided evidence of fatty degeneration. The lungs were congested, and of a somewhat bright red color. The other organs were healthy.

